# Novel Probability Density Function of Pad Asperity by Wear Effect over Time in Chemical Mechanical Planarization

**DOI:** 10.3390/ma17081817

**Published:** 2024-04-15

**Authors:** Seonho Jeong, Yeongil Shin, Jongmin Jeong, Seunghun Jeong, Haedo Jeong

**Affiliations:** Graduated School of Mechanical Engineering, Pusan National University, Busan 46703, Republic of Korea; shjeong@pusan.ac.kr (S.J.); oil5108@pusan.ac.kr (Y.S.);

**Keywords:** chemical mechanical planarization (CMP), multivariate gaussian normal distribution (MVN), asperity wear, mathematical model, contact mode, probability density function (PDF)

## Abstract

Chemical mechanical planarization (CMP) reduces film thickness, eliminates step height, and achieves high levels of planarity in semiconductor manufacturing. However, research into its mechanisms is still in progress, and there are many issues to be resolved. To solve problems in CMP, it is necessary to understand the contact phenomenon that occurs at the pad–wafer interface, especially pad asperity. Moreover, understanding the non-uniform distribution of pad asperity, such as height and radius, is essential for predicting the material removal rate (MRR). In this study, based on the existing Greenwood–Williamson (GW) theory and probability density function (PDF), a modified mathematical model that includes changes in asperity distribution was developed and validated experimentally. The contact model proposed in this study included functions that calculated the time-dependent height and radius wear of the pad asperities. Specifically, the experimentally obtained values were compared with the values obtained by the model, and the comparison results were analyzed. Thereby, it was found that the contact model and MRR model considering the change in asperity wear and distribution due to CMP proposed in this study are in better agreement with the experimental results than the existing model, which shows that the MRR can be predicted by a mathematical model using the change in asperity distribution.

## 1. Introduction

**Figure 1:**
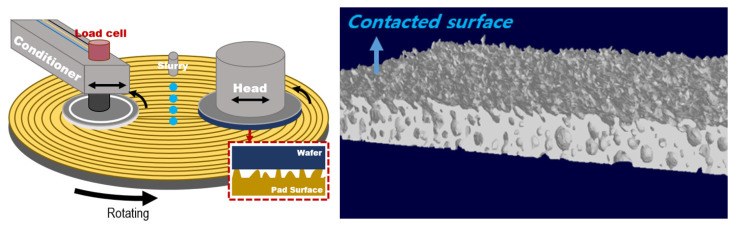
Schematic of the CMP process and 3D topography of the pad surface through micro-CT analysis.

Semiconductor processing technology has led to decreased transistor sizes and an increased number of integrated transistors, resulting in highly integrated semiconductor devices with high performance [1,2]. To integrate more devices, a multilevel interconnection structure is essential, and a surface planarization process is required for the stable deposition of the layers [3]. Chemical mechanical planarization (CMP) is a semiconductor process that enhances the quality of integrated circuits. Global planarization can solve the problem in the lithography process, wherein the depth of focus is less than the step height, and aid the development and mass production of next-generation devices [4,5]. Figure 1 depicts a schematic representation of a typical CMP process and the 3D topography of the pad surface where contact occurs with the wafer during CMP.

The pad, being a consumable, plays a significant role in mechanical removal during the CMP process. Controlling the mechanical properties and topographical conditions of the pad allows for the regulation of the CMP results [6,7,8]. Therefore, in-depth studies on the pad surface, such as contact area ratio [9,10], thermal characteristics [11,12,13], shape control [10,14], etc., are necessary.

Lee proposed a contact model for pad–wafer interfaces which included the contact deformation of asperities and mathematical models of participating particles [15]. Kim derived a physical model based on the Greenwood—Williamson (GW) contact theory, which divides the contact mode between elastic and plastic components and mathematically calculates the load transferred to abrasive particles, accounting for the contact between the pad and wafer to understand an oxidative MRR mechanism [16].

In this paper, a novel contact model considering the wear effect of the pad asperities is proposed. The improved model is validated by the experimental data. Additionally, the predictive performances of the previous analysis model are also compared with those of the proposed model in order to further validate the effectiveness of the developed model.

## 2. Theorectical Background

### 2.1. Contact Theory in CMP

Contact is the most basic element and an indispensable part in the material removal of CMP. In other words, the majority of physical actions during the process are rooted in contact interactions, such as the contact between the pad bulk and the wafer, the contact between pad asperities and the micro surface, and interactions with slurry particles. Therefore, an understanding of contact is important for analyzing the material removal mechanism. Over the decades, contact theory in CMP has been modified and developed based on the Hertzian and Greenwood–Williamson theories. Greenwood and Williamson [17] and Vasilev [18] presented a contact theory wherein the random asperity heights for a surface shape were expressed with asperities of irregular sizes and heights, and derived the material removal rate for the upper and lower areas of the pattern. In this study, we aimed to construct a model that concretely defines the random distribution of pad asperities within the pad and the associated contact phenomena, building upon existing contact theories.

#### 2.1.1. Contact: PDF of Pad Asperity

The CMP pad macroscopically has a constant shape having concentric grooves. However, at a microscopic level, the pad surface has micro-scale pad asperities distributed. In addition, these pad asperities do not have a certain shape and size and exist randomly. In the above-mentioned CMP contact models, a factor that greatly affects the results is this random asperity distribution. This is because variations in the shape or size of the pad asperities can result in different polishing results when they come into contact with the same wafer surface [19,20,21,22]. Furthermore, the conditioning process used to maintain the repeatability of polishing in the CMP process ultimately aims to control the distribution of asperities to manage the contact [23]. Therefore, it is necessary to assume and express the asperity distribution of the pad in more detail. The contact area of different asperities with the wafer surface was approximated as a circle shape with an area equal to each area. The overall shape of the pad asperity is assumed to be conical with a spherical top. These approximations are appropriate because the contact model to be presented does not consider the variety of contact geometries of asperities. Prior studies have approximated the height distribution of asperities to follow an exponential distribution, while assuming that the curvature radius of the asperities approximates a log-normal distribution, and the density function according to the height and radius is as follows [17,18]:

(1) $$\phi \left(z\right)=\frac{1}{\sigma_{z}\sqrt{2\pi }}\cdot exp\left(-\frac{z^{2}}{2{\left(\sigma_{z}\right)}^{2}}\right)\;z\geq 0$$

(2) $$\phi \left(R\right)=\frac{1}{R\sigma_{R}\sqrt{2\pi }}\cdot exp\left(-\frac{{\left(\ln R-\mu_{R}\right)}^{2}}{2{\left(\sigma_{R}\right)}^{2}}\right)\;R\geq 0$$

where *z* and $\sigma_{z}$ denote the asperity height and the deviation in asperity height, respectively, and where *R*, $\sigma_{R}$, and $\mu_{R}$ denote the asperity radius, the deviation in asperity radius, and the mean value of radius, respectively.

#### 2.1.2. Contact: Wear Affect in CMP

As shown in Figure 1, the CMP process is achieved through the relative rotation between the target wafer material and the CMP pad. The relative rotation following contact due to applied pressure generates the polishing of the wafer surface, but the same applies to the pad asperities. Many experimental observations have shown that the polish rate will drop dramatically if the pad is not conditioned [6,23,24]. Some have shown a connection between pad surface asperity height distribution and removal rate decay [25,26]. Oliver et al. [25] showed that the average asperity height in terms of roughness is continuously reduced with CMP time. Lawing’s experimental measurements [26] with the interferometry of the pad surface topography showed that the pad surface–height distribution changes in that a second peak occurs to the right of the existing distribution during the CMP process without conditioning. The second peak indicated a change in the tall asperities due to contact, and this point was described as differing from the height distribution measured through a conventional non-contact pad. However, the changes in pad asperities due to the polishing action are not limited to their height. The wear of the asperities from the relative rotation results not only in a reduction in height but also in deformation from compression. Jeong et al. [11] described that the pad asperities undergo variations not only in height but also in size and angle depending on CMP, conditioning, and process temperature, and these effects influence the MRR results. Such changes become more significant with the optimization of process conditions, making it essential to consider them in the PDF of asperities.

### 2.2. Multivariate Gaussian Normal Distribution (MVN) Theory

The multivariate normal distribution (MVN) is a fundamental concept in statistics and probability theory used to model and analyze the relationships among multiple variables. It serves as a mathematical model for the probability distribution that arises when several random variables are jointly normally distributed, and it is widely applied in various fields [27,28].

**Figure 2:**
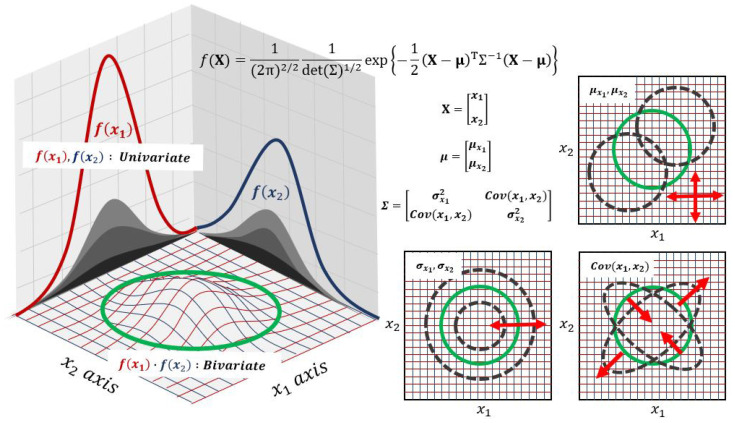
The schematic of the bivariate Gaussian normal distribution theory (one version of the multivariate Gaussian normal distribution) and distribution tendency by component (x: variable matrix, μ: mean value, σ: deviation, Cov(x): covariance) of equation.

The key characteristic of the multivariate normal distribution is its representation of data in a multidimensional space. In this study, we used bivariate normal distribution theory, which uses two variables among multivariate normal distributions. In general, elements composing the bivariate normal distribution function include variables (x), standard deviations (σ), covariances (Cov(x)), and mean values (μ). Figure 2 shows a schematic diagram of this theory, illustrating the correlation between each component and the bivariate normal distribution. The two variables each follow a univariate Gaussian normal distribution, and through the derivation process, these distributions are extended to a bivariate normal distribution. The mean values of each variable can shift the overall distribution, and the standard deviations can adjust the variance of the overall distribution. Finally, the covariance between the two variables reflects the correlation between the two univariate distributions, and it alters the shape of the bivariate distribution accordingly. The detailed derivation process and explanation are described in Section 3.2.

The theoretical background of the multivariate normal distribution is crucial for understanding and interpreting the statistical properties of multivariate data, making it applicable in multidimensional statistical analysis, pattern recognition, machine learning, data mining, and other diverse fields. It provides a foundational framework to comprehend and work with the statistical characteristics of multivariate data.

## 3. Model Development

### 3.1. Pad Asperity Evolution According to CMP Time

As mentioned above, since the asperities of the CMP pad have very random height distributions or radius distributions, it is important to express them as probability distributions. In addition, these distributions are changed by contact according to CMP time. Therefore, in this section, the PDF of asperities is proposed considering time as well as height and radius.

**Figure 3:**
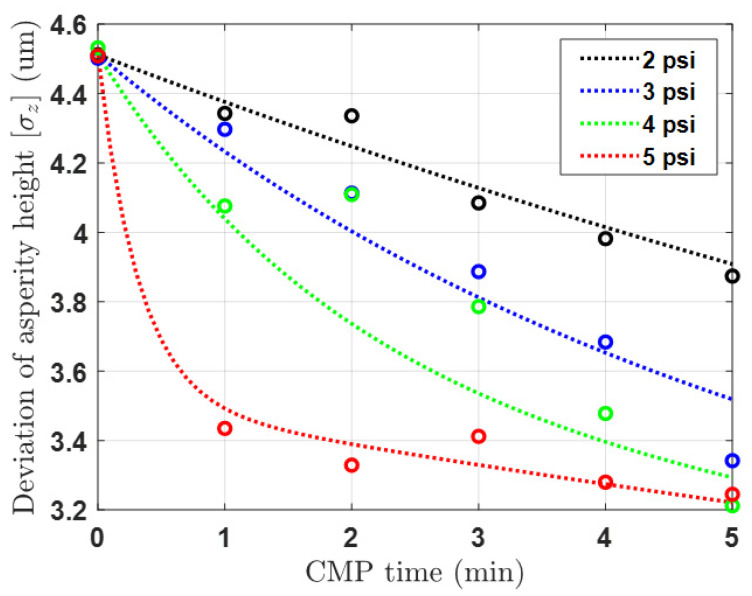
Deviation in pad asperity height after CMP according to pressure and time conditions.

**Figure 4:**
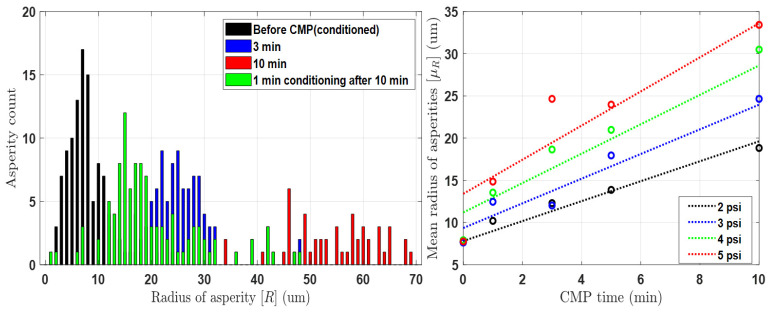
(**Left**): Distribution of pad asperity radius before and after 4 psi CMP process. (**Right**): Mean radius after CMP according to pressure and time conditions.

Figure 3 and Figure 4 depict the results of the pad surface properties after CMP an 8 inch oxide wafer. In order to detect the asperity abrasion due to pad–wafer contact, blanket wafers were polished in the absence of conditioning in 60 s steps up to 600 s of CMP time. Each graph illustrates the changes in the height distribution and radius distribution of asperities over CMP time, and the analysis was conducted using a confocal microscope and micro-CT measurements. In the case of changes in asperity height deviation as shown in Figure 3, wear occurs in asperities that can participate in CMP at heights greater than the effective asperity height (*d*). As a result, the asperity height deviation decreases, accelerating with applied pressure and CMP time. The overall decrease in asperity height reduces the ability of the asperity to polish the wafer, so it is generally managed through a conditioning process. The trend in the radius distribution in Figure 4 is similar. The height reduction due to relative rotational friction, coupled with the compression phenomenon, leads to an increase in the radius of the contacting asperities. In reality, the contact area of asperities is not circular but irregular in shape, but in this study, we calculated the radius of an equivalent circle with the same area as each shape. The analysis focuses on the variation in pad surface roughness over time at different pressures and times, and a fitting function has been determined for this analysis. Overall, the mean radius of the pad surface asperities due to wear increases over time, resulting in a smoother surface, and this trend is accelerated with an increase in pressure and time.

**Figure 5:**
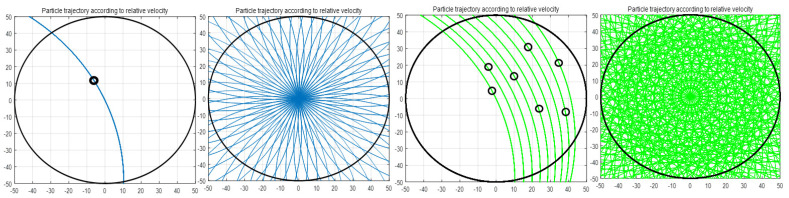
CMP trajectory simulation according to the number of contact points between the pad and wafer.

In addition, one of the asperity properties that has a great influence on CMP is the number of contacts. The number of contact points increases the probability of material removal due to the characteristics of the CMP that is relatively rotating. Figure 5 shows the result of simulating the trajectory of the contact point on the pad passing over the wafer surface during one unit of time. After calculating the relative rotation speed of the platen and head for one cycle of the contact point, MatLab coding was performed to calculate the total sliding distance of the contact point for 1 min. It can be seen that when it has multiple contact points, unlike a small number of contact points, the material removal trajectory during the unit time is large. As shown in Table 1, if CMP is continued without conditioning, there is no significant change in the number of contacts in the initial CMP, but the number of contacts on the pad surface decreases sharply the longer the CMP lasts, and the CMP efficiency at this time decreases significantly.

Therefore, it is necessary to apply these results to the model because it is possible to secure the CMP stability of the wafer by performing an optimal conditioning process and pad surface management if the changes in height, radius, and number caused by pad wear due to the above CMP are well understood. The aforementioned results were fitted using a regression model and the resulting empirical equation is as follows:

(3) $$\sigma_{z,wear}=\sigma_{z(0)}+\exp\left(\frac{-1}{\tau_{1}}\right)+\beta_{1}\cdot \exp\left(\frac{-t}{\tau_{2}}\right)-\gamma_{1}\;$$

(4) $$\mu_{R}=\left(\beta_{2}\cdot p+\gamma_{2}\right)t+\beta_{3}\cdot \exp\left(\beta_{4}\cdot p\right)$$

where $\sigma_{z(0)},\tau_{1},\tau_{2},\beta_{1}$, and $\gamma_{1}$ denote the initial deviation in asperity height and the coefficient factors related to the fitting equation of asperity deviation, and the values are 4.512, $158.01e^{-1.195p}$, 21.5, 1.411, and 2.405, respectively. Further, $\beta_{2},\beta_{3},\beta_{4}$, and $\gamma_{2}$ denote the coefficient factors related to the fitting equation of mean radius, and the values are 0.28, 5.45, 0.18, and 0.621, respectively. In the case of the pad protrusion radius, it was observed that the variation in deviation with respect to the conditions was not significant, and the overall distribution was largely dependent on changes in the mean value. Therefore, the deviation in the radius was kept fixed at its initial value in the experimental formula. The parameter coefficients of the above experimental formula can vary significantly depending on the specific pad used in the experiment. Thus, it is essential to conduct an analysis of the pad used before conducting the experiment. By substituting the experimental values into Equations (13) and (14), the asperity distribution over CMP time obtained from the experiments can be determined. Additionally, these data are utilized to derive an empirical model equation through comparison and analysis with the mathematical model equation for the time-dependent asperity distribution outlined in Section 3.2.

### 3.2. Novel PDF Model with MVN Theory

In order to develop a novel PDF model for the evolution of surface roughness that incorporates the consideration of elapsed time, it is imperative to establish a definition for the temporal rate of change in surface roughness. Borucki considered the wear rate of pad asperity during the process using the Archard equation [29,30]. The definition of the rate of change in the height of surface roughness has been established, and the formulation for the rate of change in the radius of roughness, which is considered as an additional factor in this study, adheres to the same equation and is as follows:

(5) $$\frac{dz}{dt}=-\frac{4c_{a}E}{3\pi \sqrt{R}}{\left(z-d\left(t\right)\right)}^{\frac{1}{2}},\;\frac{dR}{dt}=\frac{4c_{b}E\sqrt{z-d(t)}}{3\pi }{\left(R\right)}^{-\frac{1}{2}}$$

where *E* denotes the elastic modulus of asperity and $c_{a}$ and $c_{b}$ are parameters that are proportional to the sliding velocity of the asperity relative to the wafer. To obtain the equation for the material removal rate using the GW contact theory considering the variation in asperity height distribution, the variation in asperity radius distribution, and the asperity wear rate, a novel PDF is required. Therefore, to derive the overall density function of asperities over time considering the time-dependent changes in height and radius, the two aforementioned partial differential equations for height and radius need to be rearranged as Equations (6) and (7).

(6) $$\frac{\partial \phi (z,t)}{\partial t}-\frac{4c_{a}E}{3\pi \sqrt{R}}\cdot \frac{\partial }{\partial z}\left(\sqrt{z-d(t)}\cdot \phi \left(z,t\right)\right)=\frac{4c_{a}E}{3\pi \sqrt{R}}\cdot \frac{1}{2\sqrt{z-d(t)}}\phi \left(z,t\right)\;$$

(7) $$\frac{\partial \phi (R,t)}{\partial t}-\frac{4c_{b}E}{3\pi }\sqrt{z-d(t)}\cdot \frac{\partial }{\partial R}\left(\frac{1}{\sqrt{R}}\cdot \phi \left(R,t\right)\right)=\frac{-4c_{b}E\sqrt{z-d(t)}}{3\pi }\cdot \frac{1}{2\sqrt{R^{3}}}\phi \left(R,t\right)$$

To solve these partial differential equations, *z*, *R*, and *t* are expressed as a function of the new variable *s*, and similarly, ϕ can also be expressed as a function of *s*. Furthermore, ϕ, *z*, *R*, and *t* can be expressed as three ordinary differential equations. Equations (8) and (9) represent ordinary differential equations for calculating the partial differential equation of the asperity height, and the radius equation is also calculated through the same process.

(8) $$\frac{d\phi }{ds}=\frac{dz}{ds}\frac{\partial \phi }{\partial z}+\frac{dt}{ds}\frac{\partial \phi }{\partial t},\;\frac{d\phi }{ds}=\frac{4c_{a}E}{3\pi \sqrt{R}}\cdot \frac{1}{2\sqrt{z-d(t)}}\phi \left(z,t\right)$$

(9) $$\frac{dz}{ds}=-\frac{4c_{a}E}{3\pi \sqrt{R}}\sqrt{z-d(t)},\;\frac{dt}{ds}=1$$

The initial values are used to solve Equations (8) and (9). The assumptions that $\phi \left(s\right)=\phi_{0}\left(z_{0}\right)$ and t=0 considered the asperity height distribution before wear as a PDF used in the GW theory. Solving Equations (8) and (9), we obtained s=t and $z_{0}$. Furthermore, $R_{0}$ was obtained through the same calculation process for the asperity radius, as presented in Equation (10).

(10) $$z_{0}=z+w_{z}t\sqrt{z-d(t)}+0.25{\left(w_{z}t\right)}^{2},\;R_{0}={\left(R^{3}+3w_{R}t\sqrt{R^{3}}+\frac{9}{4}{\left(w_{R}t\right)}^{2}\right)}^{\frac{1}{3}}$$

where $z_{0}$ and $R_{0}$ denote the initial state before wear occurs, and $w_{z}$ and $w_{R}$ denote substituted parameters with values of $w_{z}=\left(4c_{a}E\right)/\left(3\pi \sqrt{R}\right)$ and $w_{R}=\left(4c_{b}E\sqrt{z-d(t)}\right)/\left(3\pi \right)$. Finally, by mathematically defining and solving each of these equations, the density function of asperities over CMP time can be expressed as follows:

(11) $$\phi \left(z,t\right)=\left[1+\frac{w_{z}t}{2\sqrt{z-d(t)}}\right]\cdot \phi_{0}\left(z_{0}\right),\;\phi \left(R,t\right)=\left[1-\frac{w_{R}t}{2\sqrt{R^{3}}}\right]\cdot \phi_{0}\left(R_{0}\right)$$

**Figure 6:**
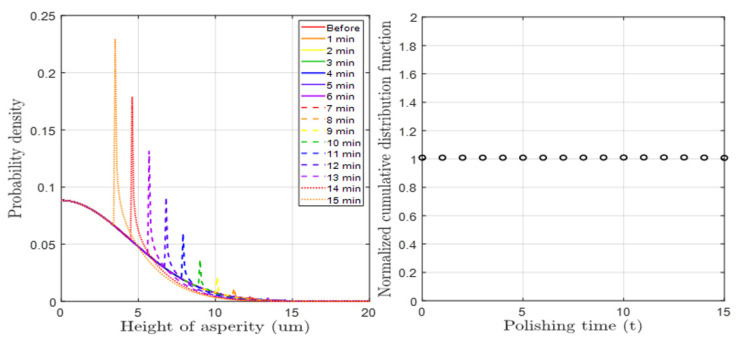
(**Left**): Simulation of time-dependent asperity height distribution. (**Right**): Sum of the probability density varied due to height wear at each CMP time.

**Figure 7:**
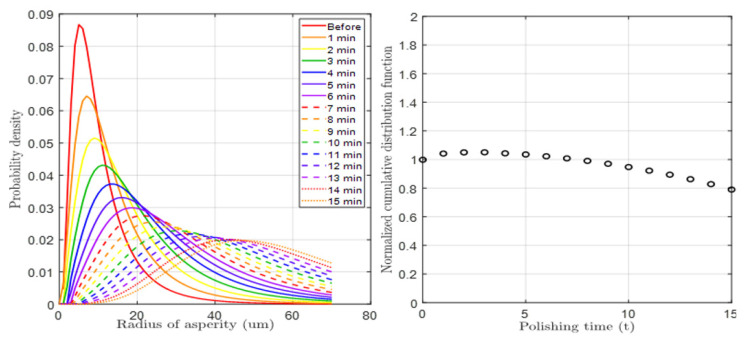
(**Left**): Simulation of time-dependent asperity radius distribution. (**Right**): Sum of the probability density varied due to radius wear at each CMP time.

Figure 6 and Figure 7 show PDF graphs considering height and radius wear, respectively, obtained through a mathematical solution process. In the case of height PDF, the height of the overall tall asperities decreases due to CMP wear, and this phenomenon gradually increases. On the other hand, in the case of radius PDF, the mean radius increased with CMP time. In both cases, it was assumed that there was no stochastic error, as the sum of the functions remained at 1 under each CMP condition. The reason for this assumption is that in a PDF, the sum of the probabilities for all variable values must be 1. However, there were conditions where the radius range set in the radius function was exceeded, but this was a setting problem and not a problem with the function itself. Furthermore, since the trends of both functions closely matched the experimentally analyzed results, their effectiveness was deemed valid.

In this study, two variables, *z* and *R*, follow a normal distribution and log-normal distribution with mean values $\mu_{z}$ and $\mu_{R}$ and standard deviations $\sigma_{z}$ and $\sigma_{R}$, respectively. The variable *R* used the logarithmic form ln(R) when constructing matrices and functions. The correlation between these two variables is denoted as Σ. The inherent irregularities in asperity height and radius do not allow for a direct linear relationship. Therefore, since *z* and ln(R) were assumed to be independent variables with a correlation of 0, Cov(z,ln(R)), a component in the Σ matrix, was also assumed to be 0. The probability vector variable x represents the rate of change of asperities over time obtained through partial differential equations, and its components consist of *z* and ln(R). The expressions of the covariance (Σ) and mean (μ) matrices for variable x are as follows:

(12) $$x=\left[\begin{array}{c} z+w_{z}t\sqrt{z-d(t)}+0.25{\left(w_{z}t\right)}^{2}\\ \ln{\left(R^{3}+3w_{R}t\sqrt{R^{3}}+\frac{9}{4}{\left(w_{R}t\right)}^{2}\right)}^{\frac{1}{3}} \end{array}\right]$$

(13) $$\Sigma =\left[\begin{array}{cc} \sigma_{z}^{2} & Cov(z,\ln(R))\\ Cov(z,\ln(R)) & \sigma_{\ln(R)}^{2} \end{array}\right],\;\mu =\left[\begin{array}{c} 0\\ \ln\left((0.28p+0.621)t+5.45\exp(0.18p)\right) \end{array}\right]$$

**Figure 8:**
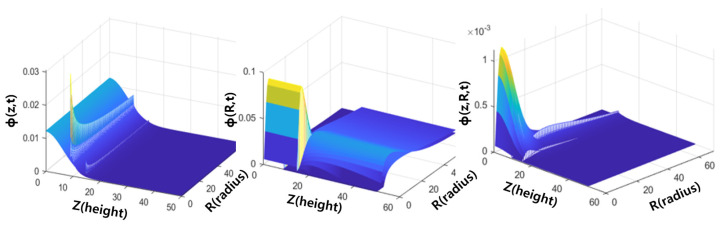
(**Left**): Probability distribution graph of asperity height according to CMP time (5, 10 min CMP conditions from Figure 6(left)). (**Middle**): Probability distribution graph of asperity radius according to CMP time (5, 10 min CMP conditions from Figure 7(left)). (**Right**): Joint probability distribution graph of asperity height and radius obtained through MVN theory (5, 10 min CMP conditions).

The equation for the distribution of asperities considering the time-dependent changes in the height and radius of the asperities is as follows:

(14) $$\phi \left(x,t\right)=\frac{1}{R2\pi \sqrt{\det(\Sigma )}}\left[1-\frac{w_{R}t}{2\sqrt{R^{3}}}\right]\left[1+\frac{w_{z}t}{2\sqrt{z-d(t)}}\right]\exp\left(-\frac{1}{2}{\left(x-\mu \right)}^{T}\Sigma^{-1}\left(x-\mu \right)\right)$$

where d(t) in the asperity density function denotes the effective asperity height between the wafer and the CMP pad. The effective asperity height varies over time to maintain load balance. Therefore, it is preferable to express d(t) as a function of time, and it can be mathematically derived since it cannot be obtained through measurements. This derivation is based on the force equilibrium equation, which asserts that the sum of contact loads on the wafer and the applied loads on the polishing head are equal. Figure 8 shows a graph for each distribution function over time. Unlike the case where only height wear or only radius wear is considered, the novel PDF proposed in this study can reflect the shape changes of asperities and their distribution in more detail. Therefore, we proposed a physical model that can predict material removal based on this novel function.

### 3.3. Material Removal Rate Model

In order to predict material removal in the context of the wafer, it is necessary to establish a novel physical model that takes into account the evolution of pad asperity distribution and the contact mode of these asperities with respect to processing time. Applying the models and functions outlined in Section 2 and Section 3, the equations for contact load, contact area, and the number of contacted asperities were redefined considering the changes in the distribution of pad asperity based on the process duration using the GW contact theory, as shown in Equations (3) and (4). This set of equations can be solved numerically with the following procedure:

1. Initialize ϕ(z,R,t) to $\phi_{0}\left(z,R\right)$ and d(t) to d(0);
2. Solve for the load-balancing separation d(t) using balance equation of force as in Equations (15) and (16);
3. Calculate the actual contact-force $F_{e,ep,p}$, the actual contact area $A_{e,ep,p}$, and the number of contact asperities $n_{e,ep,p}$ as in Equations (17)–(19);
4. Integrate the evolution equation for the PDF for one time step;
5. Calculate the material removal rate as in Equation (20);
6. Increment the time and repeat from step 2 until complete.

In detail, when the wafer comes into contact with the pad, the sum of the contact load due to the contact modes of the asperities is equivalent to the load applied to a specific region of the wafer through the head of the CMP machine. This equivalence allows the application of the force equilibrium equation, and it can be expressed as Equations (15) and (16). In addition, the pad asperity is divided into three contact modes, including elastic (*e*), elasto–plastic transition region (ep), and plastic (*p*), depending on the amount of indentation, as shown in Equations (17)–(19) [16,31,32,33].

(15) $$F_{Total}=F_{C,A}\left(t\right)\;$$

(16) $$F_{C,A}\left(t\right)=F_{e}\left(t\right)+F_{ep}\left(t\right)+F_{p}\left(t\right)\;$$

(17) $$F_{C,A}=\left\{\begin{array}{c} F_{e}:\frac{4EN(t)}{3}\int_{0}^{\infty }\int_{d(t)}^{d\left(t\right)+\delta_{Y}}\sqrt{R}\cdot {\left(z-d\left(t\right)\right)}^{\frac{3}{2}}\cdot \phi \left(z,R,t\right)dzdR\;\;\;\;0\leq \delta \leq \delta_{Y}\\ F_{ep}:\pi N\left(t\right)\int_{0}^{\infty }\int_{d\left(t\right)+\delta_{Y}}^{d\left(t\right)+\delta_{P}}R\left(z-d\left(t\right)\right)\left[H-\left(\frac{2H\ln\frac{54\delta_{Y}}{z-d(t)}}{3\ln54}\right)\right]\cdot \;\;\;\;\;\;\;\;\;\\ \;\;\;\;\left[1+3{\left(\frac{\frac{z-d(t)}{\delta_{Y}}-1}{53}\right)}^{2}-2{\left(\frac{\frac{z-d(t)}{\delta_{Y}}-1}{53}\right)}^{3}\right]\cdot \phi \left(z,R,t\right)dzdR\;\;\delta_{Y}\leq \delta \leq \delta_{P}\\ F_{p}:2\pi HN\left(t\right)\int_{0}^{\infty }\int_{d\left(t\right)+\delta_{P}}^{\infty }R(z-d(t))\cdot \phi \left(z,R,t\right)dzdR\;\;\;\;\;\delta_{P}\leq \delta \end{array}\right.$$

(18) $$A_{C,A}=\left\{\begin{array}{c} A_{e}:\pi N\left(t\right)\int_{0}^{\infty }\int_{d(t)}^{d\left(t\right)+\delta_{Y}}R\left(z-d\left(t\right)\right)\cdot \phi \left(z,R,t\right)dzdR\;\;\;\;\;0\leq \delta \leq \delta_{Y}\;\;\;\\ A_{ep}:\pi N\left(t\right)\int_{0}^{\infty }\int_{d\left(t\right)+\delta_{Y}}^{d\left(t\right)+\delta_{P}}R\left(z-d\left(t\right)\right)\cdot \;\;\;\;\;\;\;\;\;\;\;\;\\ \;\left[1+3{\left(\frac{\frac{z-d(t)}{\delta_{Y}}-1}{53}\right)}^{2}-2{\left(\frac{\frac{z-d(t)}{\delta_{Y}}-1}{53}\right)}^{3}\right]\cdot \phi \left(z,R,t\right)dzdR\;\delta_{Y}\leq \delta \leq \delta_{P}\;\;\;\;\\ A_{p}:2\pi N\left(t\right)\int_{0}^{\infty }\int_{d\left(t\right)+\delta_{P}}^{\infty }R\left(z-d\left(t\right)\right)\cdot \phi \left(z,R,t\right)dzdR\;\;\;\;\;\delta_{P}\leq \delta \;\;\; \end{array}\right.$$

(19) $$n_{C,A}=\left\{\begin{array}{c} n_{e}:N\left(t\right)\int_{0}^{\infty }\int_{d(t)}^{d\left(t\right)+\delta_{Y}}\phi \left(z,R,t\right)dzdR\;\;\;\;\;\;\;\;0\leq \delta \leq \delta_{Y}\\ n_{ep}:N\left(t\right)\int_{0}^{\infty }\int_{d\left(t\right)+\delta_{Y}}^{d\left(t\right)+\delta_{P}}\phi \left(z,R,t\right)dzdR\;\;\;\;\;\;\;\delta_{Y}\leq \delta \leq \delta_{P}\\ n_{p}:N\left(t\right)\int_{0}^{\infty }\int_{d\left(t\right)+\delta_{P}}^{\infty }\phi \left(z,R,t\right)dzdR\;\;\;\;\;\;\;\;\delta_{P}\leq \delta \end{array}\right.$$

where $F_{Total}$, *H*, and the subscript C,A denote the total force applied to the pad by machine, the hardness of asperity, and the contacted asperity, respectively. $\delta_{Y}$ is the initial yield indentation amount, which is assumed to be $\delta_{Y}=\pi^{2}RH^{2}/16E^{2}$, and $\delta_{P}$ is the approach of distant points at the onset of fully plastic asperity deformation, which is assumed to be $\delta_{P}=54\delta_{Y}$ [16]. Through the calculation process of the above equations, the equation for the effective asperity height d(t) can be derived. By utilizing this equation to solve the integral terms in the existing contact load equation, each contact load expression can be represented as a function of the total load applied to the pad by the head ($F_{Total}$). For the equation of material removal rate, the Preston equation $\left(MRR=k_{w}PV\right)$ is generally used as the governing equation [34], where $k_{p}$, *P*, and *V* denote the Preston constant, nominal pressure, and the relative rotational speed between the wafer and pad, respectively.

(20) $$MRR\left(t\right)=\frac{k_{p}V}{A_{norm}}\cdot \left[\frac{F_{e}\left(t\right)n_{e}\left(t\right)}{A_{e}\left(t\right)}+\frac{F_{ep}\left(t\right)n_{ep}\left(t\right)}{A_{p}\left(t\right)}+\frac{F_{p}\left(t\right)n_{p}\left(t\right)}{A_{p}\left(t\right)}\right]$$

In this study, the assumed elastic–plastic transition region of contact $F_{ep},A_{ep}$ and the asperity distribution equation (ϕ(z,R,t)) require numerical analysis due to the complexity of the derivations.

## 4. Results and Discussion

### 4.1. Experimental Condition for CMP

A colloidal silica-based CMP slurry was prepared for this experiment. A single-platen CMP machine (POLI-500 polisher, GnP Technology, Busan, Republic of Korea) was prepared for the experiments. SiO_2_ wafers with a diameter of 200 mm were prepared. The slurry flow rate was 150 mL/min, and pad type was IC1000. The pad mechanical properties of IC1000 were obtained from [35,36]. Pressures of 2 and 5 psi were applied and the table and carrier velocities were set to 93 rpm and 87 rpm. No conditioning between CMP processes was performed, as the focus was specifically on observing the impact of pad wear on the CMP efficiency.

### 4.2. Comparison of CMP Result and Model

To test the reliability of the material removal rate model derived using the aforementioned mathematical modeling process, its accuracy in predicting the result of the actual CMP process must be analyzed. As a comparative group to verify the consistency of the model, a model considering only the height wear of the pad asperities was applied. Therefore, to verify the consistency of the model proposed in this study, an oxide CMP experiment was conducted to compare the MRR results of the simulations with that of the experimental data. The proposed model considers multiple process elements that affect the polishing in the derivation process, based on the geometrical characteristics of the pad asperity.

**Figure 9:**
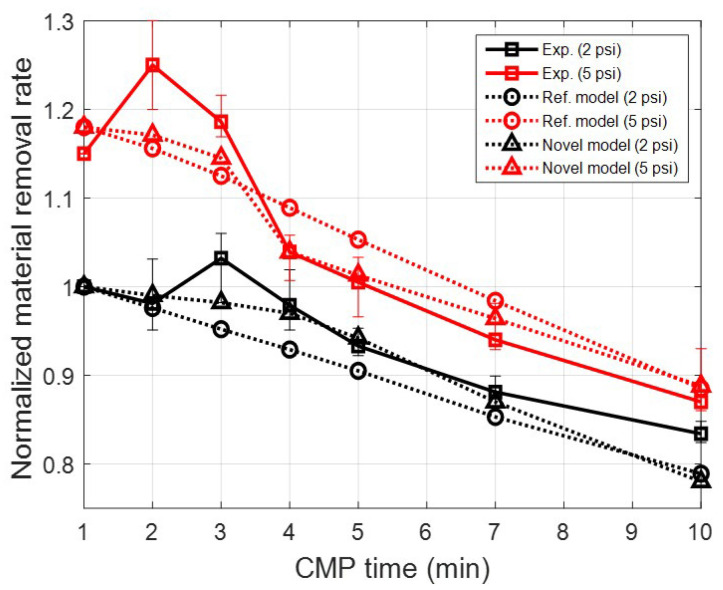
Normalized material removal rate over time under various applied pressures.

Figure 9 shows the normalized MRR result over pressure and CMP time. The initial MRR under a pressure condition of 2 psi was used as the reference for normalization. In each pressure condition, the MRR decreases with the CMP time. This is because conditioning between CMP processes does not take place, leading to the sustained wear of the pad asperity without recovery. However, in actual CMP (solid lines in Figure 9), the MRR does not show a pronounced decrease initially. Instead, it either increases or remains stable. Subsequently, as the CMP time continues and passes a certain duration, the MRR decreases rapidly. This means that the conditioning process is not necessary at every moment. Since the conditioning process ultimately consumes the life of the pad, it needs to be applied appropriately according to the condition of the asperities and the MRR results.

**Figure 10:**
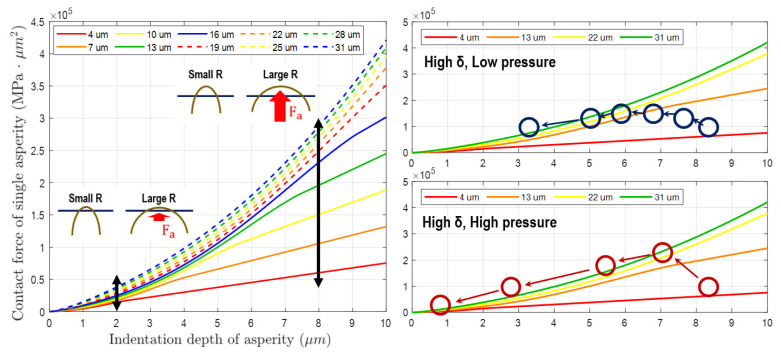
(**Left**): Contact force depending on the amount of indentation and radius size of a single asperity. (**Right**): Simulation of changes in contact force due to changes in asperity wear under high and low pressure conditions.

Figure 10 shows the contact force based on Equation (17) with respect to the indentation depth and radius of a single asperity. In the existing model that considers only the wear of the height distribution, the continuous reduction in contact force of a single asperity occurs because the asperity radius and the number of contacts are fixed. This results in a sustained decrease in material removal rate, as depicted in the graph with a circle marker in Figure 9. However, in the novel model that takes into account changes in the asperity radius distribution and contact number, the variation in contact force of a single asperity due to wear progresses differently. In Figure 10(left), The difference in contact force according to the contact radius is important when the indentation depth of the asperity is large. Typically, this phenomenon is similar to the initial state of the unworn asperities of a conditioned pad. These tall asperities not only decrease in height through CMP, as shown in Figure 6, Figure 7, and Figure 10(right), but also increase in radius. Therefore, the variation in contact force for a single asperity during CMP is as follows. During initial CMP, the asperities decrease in height due to contact wear, resulting in a decrease in contact force. However, the increase in radius due to wear compensates for the decrease in contact force. This effect is dominant in the high-indentation-depth region, and this phenomenon can also be confirmed by the large difference in contact force according to radius within the graph. However, if CMP continues without conditioning, both the overall number of contact asperities and their indentation depth decrease sharply. In this region, the effect of asperity radius on contact force is low and insignificant, as evident from the results of contact force according to radius in the graph’s low-indentation-depth region. These results impact the MRR variation, and they were confirmed in Figure 9.

In this contact model, changes in height and radius due to wear were additionally considered in the existing asperity PDF. In addition, the number of asperity contacts in a specific area was also reflected. This can explain the changes in material removal rate at low load and initial CMP compared to the existing model when compared to actual experimental results. Ultimately, by using this model, it is believed that the direction of asperity shape and distribution management can be studied and used for the optimal conditioning process related to pad life.

## 5. Conclusions

In this study, a pad–wafer contact model in the CMP process was proposed and experimentally verified. This contact model was also used to predict the material removal rate of SiO_2_ CMP. In the existing model, only the height of the PDF of the asperity was considered. In the proposed novel PDF, the radius distribution was additionally considered to sufficiently reflect the randomness of the pad asperities, and then a joint density function was developed by applying MVN theory. The wear of the height and radius of the asperity over CMP time was considered, which also affected the range of contact mode of a single asperity.

Therefore, the proposed asperity contact model better predicted the MRR changes during the initial and continuous CMP time course than existing model. In the initial stages of CMP, the actual MRR did not decrease rapidly despite the wear of the rough pad surface, and the reason for this was identified through a graph of the contact force according to the change in asperity shape over initial time. If wear due to CMP continues, the MRR rapidly decreases due to a decrease in overall height, an increase in radius, and a decrease in contact number. Therefore, the advantage of this research model over existing models is that it can predict this phenomenon. This contact model is expected to be used in various ways to predict changes in MRR from wafer to wafer during CMP and to carry out an appropriate conditioning process.

## Figures and Tables

**Table 1:** *N* (Number of contact objects on CMP pad).

	Bef.	1 min	2 min	3 min	4 min	5 min	10 min	Cond.
2 psi	109	121	103	113	97	91	56	114
3 psi	106	103	116	110	98	96	52	120
4 psi	121	118	101	99	80	82	60	110
5 psi	114	96	72	81	60	46		108

## Data Availability

Data are contained within the article.

## Abbreviations

The following abbreviations are used in this manuscript:

CMP	Chemical mechanical planarization
MVN	Multivariate normal distribution
GW	Greenwood–Williamson
MRR	Material removal rate
